# Comparison of Single-Incision Scrotal Orchidopexy Versus Standard Two-Incision Inguinal Orchidopexy in Children With Palpable Undescended Testis

**DOI:** 10.7759/cureus.24429

**Published:** 2022-04-24

**Authors:** Ali Asad, Ghulam Mustafa, Qumbar Ali Raza, Mahnoor Mehboob

**Affiliations:** 1 Pediatric Surgery, University of Health Sciences, Lahore, PAK; 2 Pediatric Surgery, Services Hospital, Lahore, PAK; 3 Pediatric Surgery, The Children's Hospital and University of Child Health Sciences, Lahore, PAK; 4 General Surgery, Jinnah Hospital, Lahore, PAK

**Keywords:** secondary ascent, scrotal hematoma, two-incision inguinal orchidopexy, single incision scrotal orchidopexy, undescended testis

## Abstract

Objective

This study compares the operative time and complications including scrotal hematoma and secondary ascent of single-incision scrotal orchidopexy with standard two-incision inguinal orchidopexy in children for the treatment of palpable undescended testis.

Methodology

An open-label, randomized clinical trial was conducted at the Department of Pediatric Surgery Services Hospital, Lahore, Pakistan, for six months from August 16, 2021, to March 15, 2022. A total number of 266 patients with palpable undescended testis aged 1-10 years were included in this study. Patients were randomized into two groups with an equal number of candidates (n = 133) using the lottery method. In group A, a single-incision scrotal orchidopexy was done while in group B, two-incision inguinal orchidopexy was done. Groups were compared for operative times and frequency of scrotal hematoma formation and secondary ascent one day and one week after the procedure, respectively.

Results

The mean age of our study children was 2.27+1.36 years; 159 (59.77%) children presented with right-sided palpable undescended testis and 107 (40.23%) children presented with left-sided undescended testis. Mean operative time in groups A and B was 25.35 ± 3.50 min and 45.45 ± 4.55 min, respectively (p < 0.0001). Scrotal hematoma occurred in three (2.3%) patients in group A and in 10 (7.5%) patients in group B. This difference was statistically significant (p = 0.047). In addition, secondary ascent occurred in four (3.0%) patients in group A and two (1.5%) patients in group B. This difference was not statistically significant (p = 0.4).

Conclusion

Single-Incision scrotal orchidopexy is simple, effective, less time consuming, and has fewer complications in terms of scrotal hematoma and secondary ascent as compared to the two-incision inguinal approach in children of palpable undescended testis.

## Introduction

The undescended testis is the most common birth anomaly in boys [1]. A true undescended testis has been arrested anywhere in the path of normal descent [2]. The management of undescended testis (UDT) depends upon the location of the testes. Different modes of treatment are available for palpable undescended testes [3]. Hormonal therapy can be useful in around 20% of the cases while 95% of cases are corrected by surgery [4]. Two incision-inguinal approach has been a gold standard surgical procedure since its introduction [5,6]. Bianchi and Squire, in 1989, described single-incision scrotal orchidopexy to reduce the risk of complications which were observed in the two-incision-inguinal orchidopexy technique [7]. Moreover, single-incision scrotal approach is also used for congenital hernia and hydrocele but it has not gained widespread acceptance for undescended testis [8].

Ramzan et al. found a significant difference in operative time but did not notice any significant difference in the complications between the two procedures. In their study, the scrotal hematoma rate was 2.2% in single-incision orchidopexy and 4.4% in two-incisions inguinal approach [2]. Another study was done by Eltayeb which showed a significant difference in complications between these two techniques. In his study, the scrotal hematoma rate was 5.7% in single-incision orchidopexy and 0.0% in two-incisions inguinal approach while the rate of secondary ascent was 5.7% in single-incision orchidopexy and 2.8% in two-incisions inguinal approach [9]. As there is considerable debate regarding the safety of this approach, this research is proposed to evaluate the safety and efficacy of single-incision orchidopexy over the standard two-incisions orchidopexy for palpable undescended testis in our institute.

## Materials and methods

Study design

An open-blinded randomized controlled trial was performed at the Department of Paediatric Surgery, Services Hospital Lahore, Pakistan, for six months. A sample size of 266 (133 in each group) patients was calculated at a 5% level of significance and 80% power of test. The expected frequency was 5.7% after single-incision scrotal orchidopexy and 0% after two-incision inguinal orchidopexy.

Inclusion and exclusion criteria

All male children with a diagnosis of palpable undescended testis in the age group of 1-10 years were included in this study. Patients suspected to have disorders of sex differentiation, retractile testis, previous history of inguinal surgery, ectopic testis, and peeping testis were excluded.

Data collection

After obtaining approval from the ethical committee of the hospital (#IRB/2021/856/SIMS), all patients fulfilling the inclusion criteria were admitted to the pediatric surgery inpatient department of Services Hospital, Lahore. Complete demographic information (name, age, sex, and address) was recorded in performa. Patients were randomized into two groups with an equal number of candidates (n = 133) using the lottery method. In group A, single-incision scrotal orchidopexy was done while in group B, two-incision inguinal orchidopexy was done.

**Figure 1:**
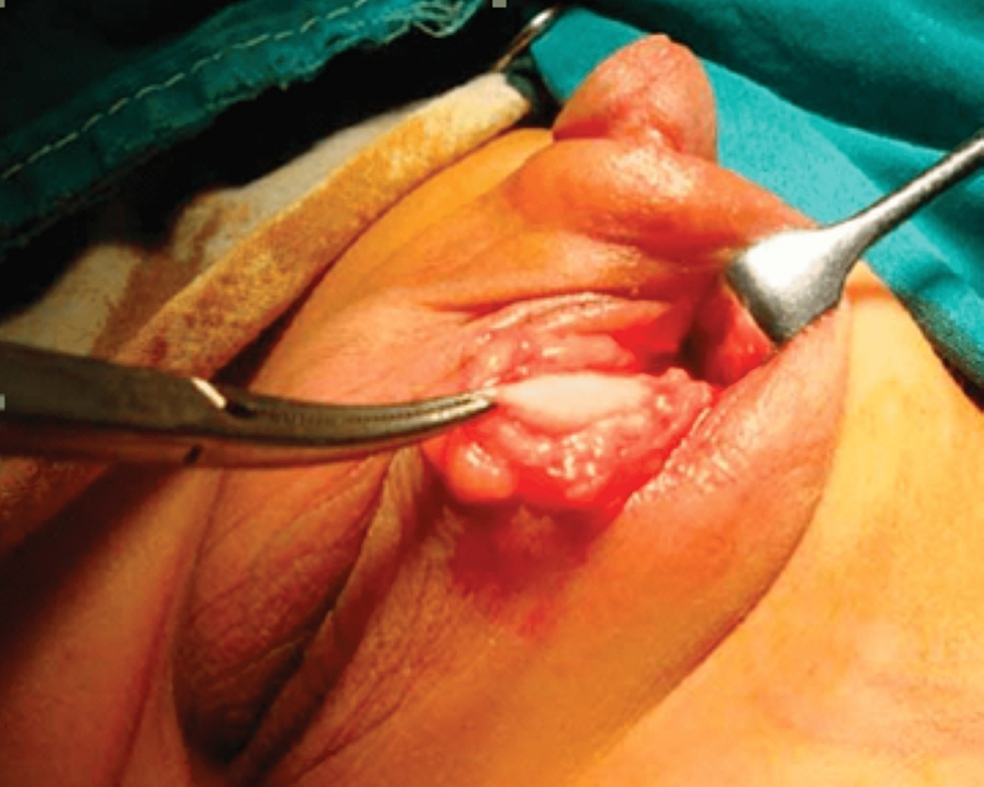
Scrotal incision and mobilization of testis

In group A, orchidopexy was performed through a single high scrotal incision. The scrotum was opened and retraction was made to visualize the testis as shown in Figure 1. Patent processus vaginalis was separated off the cord structures and ligated. Testis was mobilized into the scrotum and fixed in the dartos pouch.

**Figure 2:**
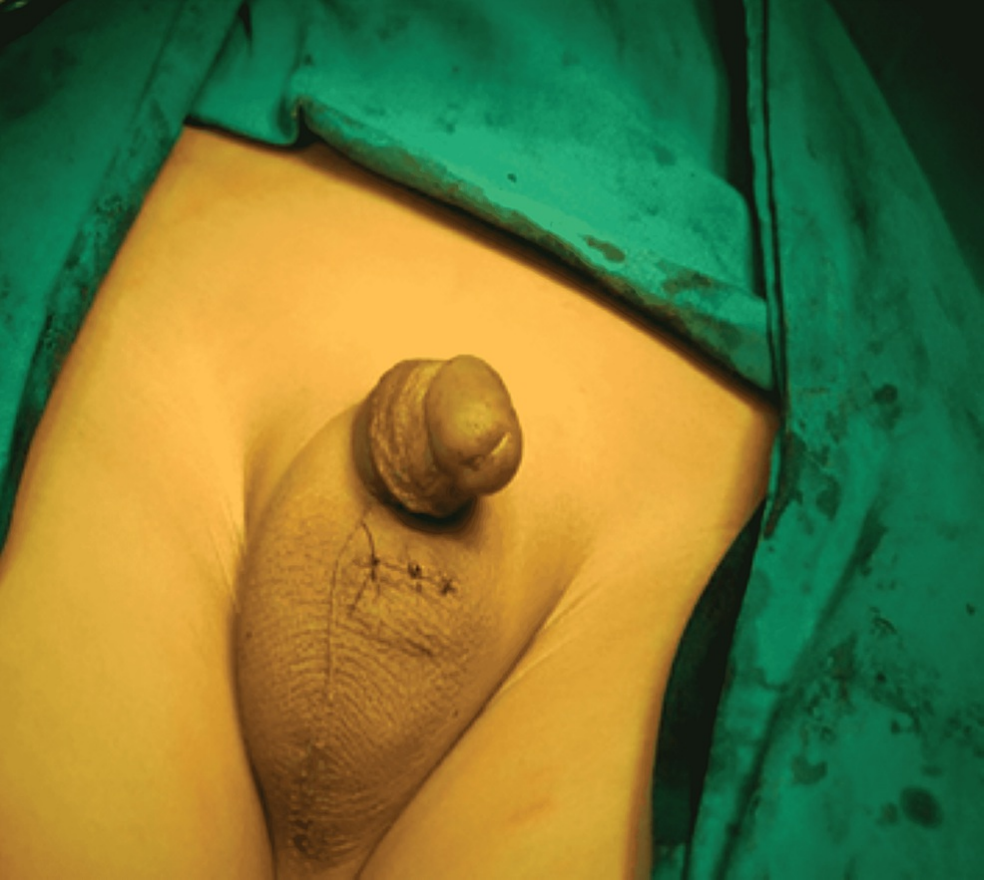
Final appearance after closure of scrotal skin incision

The final appearance of the scrotum after closure of skin is shown in Figure 2. In group B, the standard two-incision inguinoscrotal approach was used. Testis and spermatic cord were mobilized after opening the inguinal canal and brought down into the scrotum after doing a herniotomy. The sub-dartos pouch was created through a scrotal incision and the testis was fixed there. In patients with bilateral palpable undescended testes, only one side was operated and the right side was operated priorly. Operative time of the procedure was recorded. Scrotal hematoma formation was noted one day after surgery and secondary ascent was noted one week after the procedure. All the gathered information was noted on a predesigned performa.

Data analysis procedure

Data analysis was carried out using SPSS v23.0 (Armonk, NY: IBM Corp.). Age was presented as mean and standard deviation. Frequency and percentages were calculated for age groups, side, location of undescended testis, scrotal hematoma formation, and secondary ascent rate. The Student's t-test and chi-square test were applied for data analysis in both groups. Effect modifiers like age, side, and location of testis were controlled by stratification. Post-stratification chi-square test was applied taking p-value <0.05 as significant.

## Results

Our study included 266 children with undescended testis. The mean age of the patients was 2.27+1.36 years; 159 (59.77%) children presented with right-sided palpable undescended testis and 107 (40.23%) children presented with left-sided undescended testis. A comparison of baseline participant characteristics (age, gender, and location of testis) between the two groups is shown in Table 1.

**Table 1:** Baseline characteristics of participants

Demographic data	Group A (133)	Group B (133)	p-Value
Location			
External ring	62	51	0.17
Canalicular	71	82	
Age group			
< 2 years	51	56	0.53
> 2 years	82	77	
Side			
Right	79	80	0.90
Left	54	53	

Mean operative time in groups A and B was 25.35 ± 3.50 min and 45.45±4.55 min, respectively (p < 0.0001). Scrotal hematoma occurred in three (2.3%) patients in group A and 10 (7.5%) patients in group B which was statistically significant (p = 0.047). In addition, secondary ascent occurred in four (3.0%) patients in group A patients and in two (1.5%) patients in group B patients. This difference was not statistically significant (p = 0.4) as shown in Table 2.

**Table 2:** Comparison of scrotal hematoma and secondary ascent between the groups

Scrotal hematoma	Group A	Group B	p-Value
Yes	3 (2.3%)	10 (7.5%)	0.047
No	130 (97.7%)	123 (92.5%)	
Secondary ascent			
Yes	4 (3.0%)	2 (1.5%)	0.40
No	129 (97.0%)	131 (98.5%)	

Stratification of children on the basis of age and location of the undescended testis to determine their effects on the frequency of scrotal hematoma and secondary ascent rate between the groups are shown in Table 3. No patient undergoing the single-incision orchidopexy required conversion to an inguinal approach to get a tension-free orchidopexy.

**Table 3:** Stratification of location of testis and age on the frequency of scrotal hematoma and secondary ascent between the groups

Location of testis	Scrotal hematoma		Group A	Group B	p-Value
External ring	Yes		1	2	0.44
	No		61	49	
Canalicular	Yes		2	8	0.08
	No		69	74	
Secondary ascent					
External ring	Yes	2		0	0.65
	No	60		51	
Canalicular	Yes	2		2	0.88
	No	69		80	
Age groups					
< 2 years	Yes	1		3	0.35
	No	50		53	
> 2 years	Yes	2		7	0.07
	No	80		70	
Secondary ascent					
< 2 Years	Yes	1		0	0.10
	No	50		56	
> 2 Years	Yes	3		2	0.70
	No	79		75	

## Discussion

The management of an undescended testis has not changed much for all varieties of undescended testes including testes palpable distal to the inguinal canal [10]. It involves a groin incision to access the spermatic cord structures, high ligation of the processus vaginalis, and a second scrotal incision to place the testis in a dependent sub-dartos pouch without undue tension. This approach is still being practiced with varying degrees of modifications including retroperitoneal mobilization of the testicular vessels such that a new and straighter course towards the scrotum allows additional testicular descent [11].

The majority of the undescended testes are palpable distal to the inguinal canal. Furthermore, in the pediatric age group, the inguinal canal is short, with the internal and the external rings almost superimposed on one another. This coupled with the relative mobility of the skin in the inguinal region allows retraction of the skin incision, thereby enabling dissection through the scrotum without opening the inguinal canal. These facts led other workers to believe that single scrotal orchidopexy rather than two incisions may be adequate for orchidopexy in patients with a palpable, low-lying testis [12]. In 1989, Bianchi and Squire developed single-incision high scrotal orchidopexy (trans-scrotal) after observing that most palpable undescended testes were held by a shortened processus vaginalis. The technique has the advantage of less dissection, disruption of tissues, and greater comfort for day-case children [7].

In our study, the location of testis was at an external ring in 42.48% of children and canalicular in 57.52% of children. In the study of Al-Mandil et al., the location of testis was at an external ring in 40.5% of children and 59.5% of children were at the canalicular level [13]. In our study, 59.77% of children were presented with right-sided undescended testis, and 40.23% of children presented with left-sided undescended testis. Al-Mandil et al. also found a similar prevalence of side of undescended testis in their study children [13].

Our study showed that scrotal hematoma occurred in three (2.3%) patients who underwent single-incision scrotal orchidopexy and in 10 (7.5%) patients who underwent two-incision inguinal orchidopexy. A study carried out in the Colorado School of Medicine showed the results that hematoma occurred in one out of 85 patients (1.17%) for scrotal-incision orchiopexies. There was another study conducted by Ramzan et al. where scrotal hematoma occurred in 2.2% of patients in the single incision orchidopexy group and 4.4% of patients in the two-incision inguinal approach [9].

Our study has the results of secondary ascent which occurred in four (3.0%) patients in single-incision scrotal orchidopexy patients and two (1.5%) patients in the two-incision inguinal orchidopexy group. In the study of Ramzan et al., secondary ascents occurred in 2.2% of patients in single incision scrotal orchidopexy and 1.4% of patients in the two-incision inguinal orchidopexy group. The study of Al-Mandal et al. depicts secondary ascents in 1.6% of patients in single incision scrotal orchidopexy and 1.9% of patients in the two-incision orchidopexy group. Whereas, Cloutier et al. did not find any incidence of secondary ascent rate in their study patients. These authors used the same techniques of orchidopexy as used in our study [14]. This study had some limitations as important other variables such as pain assessment, hospital stay, wound infection, and testicular atrophy during the long follow-up were not considered.

## Conclusions

Based on these results, it was concluded that single-incision scrotal orchidopexy is associated with shorter operative times and has less number of complications including scrotal hematoma and secondary ascent. This technique is safe and effective for palpable undescended testis in inguinal canal or at the level of the external ring.
